# Case of Ileus

**Published:** 1847-10

**Authors:** 

**Affiliations:** Lemberg


*Case of Ileus.—A portion of Intestine Discharged by Stool.* By Dr. Nagel of Lemberg.—K. J. a domestic, aged 21, robust, always enjoying good health, except frequent attacks of colic within the last few years, was attacked in the night, 12th—13th February, 1843, with violent pains in the lower part of the abdomen, accompanied with shivering, frequent vomiting, and purging. On admission into the hospital on the morning of the 13th, he was in the following state:—Head hot and painful; tongue foul; thirst; abdomen swollen and tender to the touch; skin dry: pulse full, hard, and frequent; vomiting, with watery stools tinged with blood. (Antiphlogistic treatment.)

The symptoms continued much the same till the 16th, when they diminished in intensity, and the stools were no longer tinged with blood. On the 19th there was violent tenesmus, accompanied on the 23d with prolapsus of a portion of intestine, which, however, was easily reduced without causing pain.

On the 26th, the patient free from fever, and altogether in a satisfactory state, passed by stool a portion of intestine twenty inches long, and at some points two inches broad; it consisted of a portion of the ileum, the cœcum, appendix vermiformis, the whole of the ascending colon, and a portion of the transverse. The mucous membrane was everted, of a brownish colour striated with black, especially the cœcum; it was soft and easily removed. The peritoneal coat was likewise of a brown colour, and corroded, leaving bare the muscular coat, which was also destroyed at some points. For some days after there was slight pain at the lower part of the abdomen; but on the 23d March, the patient left the hospital perfectly cured.—*Dub. Med. Press,, from Oesterreichische Med. Wochen and Med. Gaz.*

